# Epigenetic Regulation of Trk Receptors and Neurotrophic Signalling in Neuroblastoma: Mechanisms, Plasticity, and Therapeutic Opportunities

**DOI:** 10.3390/ijms27073238

**Published:** 2026-04-02

**Authors:** Carlotta Siddi, Jihane Balla, Paola Fadda, Simona Dedoni

**Affiliations:** 1 Department of Biomedical Sciences, Division of Neuroscience and Clinical Pharmacology, University of Cagliari, 09042 Cagliari, Italy; carlotta.siddi@unicam.it (C.S.); paola.fadda@unica.it (P.F.); 2 Department of Medical Science and Public Health—DSMSP, University of Cagliari, 09042 Cagliari, Italy; 3 Neuroscience Institute, National Research Council of Italy (CNR), 09142 Cagliari, Italy

**Keywords:** neuroblastoma, Trk receptors, epigenetic regulation, MYCN, cell-state plasticity, adrenergic–mesenchymal transition, EZH2, HDAC inhibitors, tumour microenvironment, targeted therapy

## Abstract

Neuroblastoma (NB) represents a paradigmatic developmental malignancy in which lineage specification, oncogenic signalling, and epigenetic regulation converge to define tumour behaviour. Among the molecular axes shaping NB heterogeneity, neurotrophin receptors of the tropomyosin receptor kinase (Trk) family (TrkA, TrkB, and TrkC) and the p75NTR occupy a central position at the intersection between neuronal differentiation programs and malignant plasticity. While high TrkA and TrkC expression is associated with adrenergic identity, differentiation competence, and favourable clinical outcome, TrkB, frequently sustained by BDNF-driven autocrine loops, characterises mesenchymal-like, therapy-resistant states enriched in metabolic and inflammatory adaptations. Importantly, in NB, the dysregulation of neurotrophin signalling rarely arises from recurrent genetic alterations of neurotrophic tyrosine receptor kinase (*NTRK*) loci. Instead, Trk receptor expression is dynamically shaped by promoter methylation, polycomb repressive complex 2/Enhancer of Zeste homolog 2 (PRC2/EZH2)-dependent chromatin repression, MYCN-driven transcriptional silencing, enhancer rewiring, and microRNA-mediated control. These epigenetic mechanisms govern reversible transitions along the adrenergic–mesenchymal (ADRN–MES) continuum, enabling tumour cells to adapt to microenvironmental and therapeutic stress. Single-cell and spatial multi-omics approaches have further revealed that Trk-associated phenotypes are embedded within complex regulatory circuits integrating receptor tyrosine kinase (RTK) networks, cytokine signalling, metabolic remodelling, and stromal reinforcement. Here, we provide a comprehensive synthesis of the epigenetic and microenvironmental mechanisms regulating neurotrophin receptors in NB, with particular emphasis on how chromatin plasticity and cell-state transitions reshape Trk-dependent signalling outputs. We discuss advanced three-dimensional and organoid-based models that recapitulate niche-specific regulation of the Trk axis and evaluate emerging therapeutic strategies combining epigenetic modulators, differentiation-inducing agents, and RTK-targeted compounds. Understanding the temporal and spatial dynamics of Trk signalling may open new opportunities to therapeutically stabilise differentiation states and disrupt adaptive resistance programs in high-risk NB.

## 1. Introduction

The neurotrophin–Trk axis has emerged as a paradigmatic example of how developmental signalling pathways are reappropriated in cancer, providing a bridge between neuronal differentiation programs and oncogenic rewiring [1,2,3]. Neurotrophins, such as nerve growth factor (NGF), brain-derived neurotrophic factor (BDNF), and neurotrophin-3 (NT-3), orchestrate neuronal survival and lineage commitment through the activation of tropomyosin receptor kinase (Trk) receptors TrkA (encoded by neurotrophic tyrosine receptor kinase 1; *NTRK1*), TrkB (encoded by *NTRK2*), and TrkC (encoded by *NTRK3*), together with the low-affinity receptor p75NTR (encoded by nerve growth factor receptor; *NGFR*) [3,4]. Although originally described in embryonic neural development, these pathways have become increasingly relevant in oncology, particularly in neuroblastoma (NB), a neural crest-derived tumour in which neurotrophin receptors act as dynamic regulators of lineage identity, plasticity, metastatic behaviour, and therapeutic response [5,6,7]. Recent genome-wide and multi-omics approaches, including promoter methylation profiling, ATAC-seq chromatin accessibility maps, single-cell RNA sequencing, enhancer-rewiring analyses, and spatial transcriptomics, have placed Trk receptors at the core of NB regulatory architecture, linking their expression to super-enhancer activity, transcription factor circuits, and tumour–stroma crosstalk [1,2,6,7,8,9,10,11,12,13]. NB remains the most common extracranial solid tumour of childhood, accounting for 6–10% of paediatric cancers and 15% of cancer-related mortality [14,15,16]. Its biological spectrum extends from spontaneously regressing tumours to MYCN-amplified metastatic disease, a heterogeneity shaped by the interplay of genetic lesions, chromatin structure, and microenvironmental pressure [11,12,13,14,15,16,17,18,19]. Although *MYCN* amplification and *ALK* mutations remain central prognostic markers, it is now clear that epigenetic states, enhancer topology, and transcription factor circuitry are equally decisive in defining NB identity, particularly in mediating transitions along the adrenergic/noradrenergic (ADRN) to mesenchymal (MES) continuum, which shift tumour cells from differentiated, neuron-like states towards more migratory, stress-adapted, and therapy-resistant phenotypes that influence progression and treatment response [17,18,19]. Neurotrophin receptors lie at the intersection of these processes. High TrkA expression, identified by Nakagawara and colleagues, marks favourable NB subsets characterised by neuronal differentiation potential and spontaneous regression [20,21]. Foundational developmental studies also identified TrkA as the prototypical NGF receptor [22]. Conversely, TrkB, often sustained by autocrine BDNF loops, supports aggressive phenotypes through pro-survival, pro-angiogenic, and chemoresistance pathways [5,23,24]. In this context, BDNF/TrkB signalling has been shown to promote invasion and therapeutic resistance through multiple downstream effectors [24]. TrkC activation promotes differentiation and correlates with improved prognosis [18,25]. On the other hand, p75NTR provides an additional layer of modulation by acting as a context-dependent regulator of neurotrophin signalling when complexing with Trk receptors, fine-tuning their ligand-dependent pathways, while, in association with sortilin, mediating proneurotrophin-induced apoptotic signalling [26,27].

Multiple studies suggest that neurotrophin receptor expression in NB, including Trk receptors, is primarily regulated by dynamic epigenetic and post-transcriptional mechanisms rather than stable genetic alterations [13,18,28,29,30,31]. In high-risk tumours, *MYCN*-driven chromatin remodelling together with microRNA regulatory networks modulates differentiation-associated signalling pathways, including those mediated by Trk receptors, thereby sustaining malignant phenotypes [32,33,34]. In contrast, favourable NB subsets tend to preserve epigenetically permissive states that support Trk receptor expression and downstream signalling linked to neuronal differentiation and improved clinical outcome.

Consistent with the dynamic regulation of Trk receptor expression in NB, the clinical efficacy of highly selective TRK inhibitors, such as larotrectinib and entrectinib in *NTRK* gene-fusion-positive cancers, highlights the translational potential of targeting neurotrophin signalling [4,35,36]. In NB, however, canonical *NTRK* gene fusions are rare, and Trk receptor function is predominantly regulated by epigenetic mechanisms and microenvironmental cues rather than by stable genetic alterations [7,9,10,37]. In this context, more tailored therapeutic strategies for NB should aim to integrate differentiation-inducing agents, epigenetic modulators, and TRK-targeted compounds to exploit transient therapeutic vulnerabilities emerging during tumour state transitions.

## 2. Trk Receptors and Neurotrophic Pathways in Neuroblastoma

### 2.1. Neurotrophin–Trk Signalling Architecture and Network Integration in Neuroblastoma

In NB, neurotrophin receptors do not simply replay a developmental program—they assemble a modular signalling system that connects lineage history with the current tumour-state configuration. TrkA, TrkB, TrkC, and the low-affinity receptor p75NTR form context-specific signalling units in which kinase activity, co-receptor composition, and ligand availability are interpreted against a background of oncogenic lesions, chromatin accessibility, and stromal inputs [3,5,38]. These receptors can no longer be viewed as static markers of “favourable” versus “unfavourable” disease. Instead, they participate in a continuum of states in which expression levels, splice isoforms, and downstream wiring adapt as cells move along the ADRN–mesenchymal-like (MES) axis [2,6,7,8,39].

Single-cell multi-omics, super-enhancer mapping, and spatial transcriptomics indicate that *NTRK1*, *NTRK2*, and *NTRK3* reside within distinct regulatory chromatin domains that become activated or repressed as tumours undergo cellular state transitions [2,6,7,8,39]. ADRN/NOR programs are enriched for *PHOX2B*, *GATA3*, and *HAND2* associated with open chromatin and super-enhancers at *NTRK1* and *NTRK3* [8,14,40], whereas MES conversion is accompanied by enhancer rewiring, closing of ADRN loci, and BRD4-bound super-enhancers at *NTRK2* [7,8,34,41]. Within this architecture, neurotrophin pathways behave as dynamic translators of cell-state plasticity, integrating signals from oncogenic drivers like MYCN and ALK or from hypoxia and inflammatory cytokines to bias NB cells towards differentiation, survival, or invasive, therapy-resistant phenotypes [6,7,8,34,39,40,41].

At the signalling level, Trk receptors operate as high-affinity tyrosine kinase hubs. Ligand binding induces receptor dimerisation and autophosphorylation of key tyrosine residues in the intracellular domain, creating docking sites for adaptor proteins, such as SHC, FRS2, and PLCγ1 [3]. These complexes couple TrkA/B/C to canonical Ras-RAF-MEK-ERK and PI3K-AKT-mTOR cascades, as well as to PLCγ1–IP_3_–Ca^2+^/PKC, JAK/STAT3, and Rho family GTPases that regulate cytoskeletal dynamics and migration [3,25,26]. Through these axes, Trk signalling modulates transcription factors, including CREB and ELK1, regulates FOXO-dependent survival programmes, modulates autophagic flux, and influences the balance between pro-apoptotic (BAD and BIM) and anti-apoptotic (BCL-2, MCL-1, and BCL-xL) effectors [3,25,26].

Network-level phosphoproteomics places Trk receptors in the same tyrosine-kinase clusters as ALK, MET, RET, and IGF-1R, with convergent regulation of MYCN stability, FAK activity, and adhesion signalling [39,42]. These studies highlight compartmentalised signalling from endosomes and lipid rafts and identify Trk-containing modules that integrate growth factors, integrins, and cytoskeletal adaptors [42]. In parallel, proteogenomic surfaceome profiling has revealed NB-specific receptor constellations in which NTRK receptors coexist with potential immunotherapeutic targets, such as DLK1, emphasising their accessibility for antibody-based and CAR-T strategies [38,43].

Protein tyrosine phosphatases (PTPs) further refine these networks by modulating phosphorylation dynamics and pathway crosstalk, underscoring that the functional output of neurotrophin signalling in NB results from a balance between kinase and phosphatase activities rather than from Trk receptors acting in isolation [44]. Collectively, these data support a model in which neurotrophin–Trk pathways are embedded within broader RTK and phosphatase networks that control NB cell identity, plasticity, and treatment response [2,6,7,8,9,39,42,44].

### 2.2. Receptor-Specific Roles of TrkA, TrkB, TrkC, and p75NTR in Cell-State Plasticity

#### 2.2.1. TrkA/*NTRK1*: Lineage Fidelity, Differentiation Competence, and Dependence-Receptor Behaviour

TrkA/*NTRK1* is considered the most reliable marker of ADRN identity and differentiation capacity in NB, acting as a transcriptional and epigenetic marker of favourable biology [4,5,8,14,17,25,40]. High TrkA expression reflects an open chromatin configuration at the *NTRK1* locus, enriched for ADRN lineage determinants (*PHOX2B*, *GATA3*, and *HAND2*) and associated super-enhancers, together with promoter hypomethylation and reduced H3K27me3 deposition [8,14,30,40]. These features consistently position NTRK1 within NOR/ADRN clusters in single-cell and ATAC-seq studies, where its expression tracks with neuronal differentiation trajectories and spontaneous regression [1,8,14,17,39,40].

Upon NGF stimulation, TrkA preferentially induces sustained ERK1/2 activation, in contrast to the transient ERK signals typical of proliferative states, together with PI3K-AKT and PLCγ1 pathways [3,25,26]. This signalling profile drives induction of p21Cip1 and p27Kip1, cytoskeletal remodelling, neurite extension, and terminal differentiation programs reminiscent of sympathetic neuron maturation [3,25]. The duration and amplitude of ERK activation are critical: prolonged ERK activity favours differentiation and cytostasis, whereas shorter pulses promote mitogenic responses [3,25]. Phosphoproteomic datasets place TrkA within kinase subnetworks that stabilise differentiation rather than proliferation, reinforcing its role as a driver of growth arrest and maturation in *NTRK1*-high NB cells [25,39,42].

**Figure 1:**
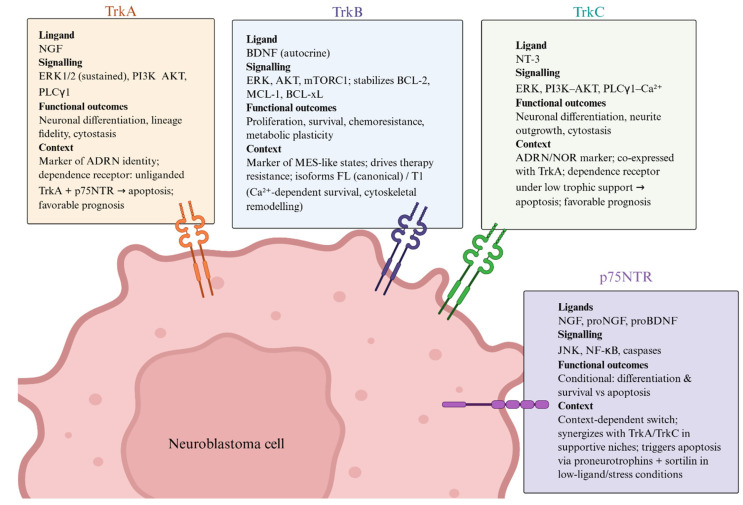
Functional architecture of neurotrophin receptors in neuroblastoma. TrkA is shown in orange, whereas TrkB and TrkC are showed in purple and green, respectively. These receptors are high-affinity receptor with tyrosine kinases enzymatic activity. In contrast, p75NTR, shown in purple, differs structurally and functionally from the Trk receptors. The figure aims to provide a comparative overview of TrkA, TrkB (FL and T1 isoforms), TrkC, and p75NTR signalling in NB. TrkA and TrkC are associated with ADRN identity and differentiation competence, whereas TrkB characterises MES-like, therapy-resistant states sustained by BDNF-driven autocrine signalling. p75NTR acts as a context-dependent modulator and dependence receptor, integrating trophic availability and stress conditions. **Abbreviations:** Trk, tropomyosin receptor kinase; p75NTR, p75 neurotrophin receptor; NGF, nerve growth factor; BDNF, brain-derived neurotrophic factor; NT-3, neurotrophin-3; proNGF, pro–nerve growth factor; proBDNF, pro–brain-derived neurotrophic factor; ERK1/2, extracellular signal-regulated kinases 1 and 2; PI3K, phosphoinositide 3-kinase; AKT, protein kinase B; PLCγ1, phospholipase C gamma 1; mTORC1, mechanistic target of rapamycin complex 1; MCL-1, myeloid cell leukemia 1; BCL-2, B-cell lymphoma 2; BCL-xL, B-cell lymphoma-extra large; JNK, c-Jun N-terminal kinase; NF-κB, nuclear factor kappa B; Ca^2+^, calcium ion; ADRN, adrenergic; MES, mesenchymal; NOR, noradrenergic; LF, late-firing; T1, transient 1; RTK, receptor tyrosine kinase.

A distinctive property of TrkA is its dependence-receptor behaviour, which is directly relevant to spontaneous tumour regression. In the absence of sufficient NGF, unliganded TrkA forms complexes with p75NTR that activate JNK and p38 MAPK pathways, promote mitochondrial cytochrome-c release, and restore apoptotic competence [26,41]. Early clinical and biological studies linking high TRKA expression to favourable outcome and regression in stage 4S NB stem from this property, whereby ligand deprivation in specific stromal contexts biases cells towards apoptosis rather than differentiation [4,14,20,26,41]. Proneurotrophins, such as proNGF, signal via p75NTR-sortilin complexes to further amplify JNK-mediated apoptosis, adding a second layer of context-dependent cell-fate control [26,27]. In addition, converging evidence indicates that the TrkA-high phenotype in NB is driven by coordinated epigenetic and microenvironmental regulation rather than intrinsic genetic programs [18,28,30,40,45], since high-risk tumours enforce MYCN-dependent chromatin repression and PRC2/EZH2-mediated silencing of differentiation-associated loci, while favourable phenotypes retain hypomethylated, enhancer-accessible NTRK1 states that support differentiation [18,30]. Stromal cues further reinforce these programs, with Schwannian-rich, ECM-supported niches promoting TrkA/TrkC-associated differentiation and inflammatory or hypoxic regions favouring TrkB-high, mesenchymal-like states [4,9,14,17,20]. Taken together, these findings position TrkA as more than a passive prognostic marker: it is a lineage-defining receptor whose activity integrates chromatin architecture, ligand availability, and stromal cues to determine whether NB cells differentiate, survive, or undergo apoptosis [3,4,5,8,14,17,20,25,26,30,40,45] (Figure 1).

#### 2.2.2. TrkB/*NTRK2*: MES Transition, Metabolic Plasticity, and Therapy Resistance

TrkB/*NTRK2* is tightly associated with aggressive, therapy-resistant NB, largely via BDNF-driven autocrine loops. TrkB is almost invariably co-expressed with its ligand BDNF, forming a potent autocrine survival circuit that sustains proliferation, angiogenesis, metabolic remodelling, and chemoresistance [5,21,24]. Canonical TrkB signalling drives AKT, ERK, and mTORC1 activation, stabilises anti-apoptotic proteins (BCL-2, MCL-1, and BCL-xL), and supports survival under oxidative and metabolic stress [5,21,24]. A central feature of TrkB-dependent malignancy is metabolic plasticity. MYCN and HIF-1α cooperatively reinforce *NTRK2* transcription, creating a feed-forward loop that couples neurotrophin signalling to glycolysis, lactate production, and VEGF-mediated angiogenesis [21,41,46]. TrkB-high NB cells exhibit increased glutamine dependency and enhanced fatty-acid oxidation, supporting growth in nutrient-limiting or hypoxic conditions [21,41,46]. Through this deep integration with metabolic circuitry, TrkB imposes a malignant program highly adaptive to environmental and therapeutic stressors [5,21,24,41,46]. A major conceptual refinement in NB biology is the tight link between TrkB expression and the ADRN–MES axis. MES states, characterised by inflammatory signatures, cytoskeletal remodelling, and high migratory capacity, are intrinsically more resistant to chemotherapy and targeted agents [2,6,7,8,39]. Multi-omics analyses demonstrate that *NTRK2* transcription increases during transitions towards MES identity, driven by BRD4-occupied super-enhancers that stabilise TrkB expression while repressing ADRN lineage factors, such as PHOX2B and HAND2 [6,7,8,34,39]. Single-cell trajectory analyses reveal that enhancer rewiring and chromatin closing at ADRN loci accompany *NTRK2* upregulation, positioning TrkB as both a marker and driver of therapy-resistant MES phenotypes [6,7,8,18,34,39,40].

In this setting, TrkB does not operate in isolation. Network-based phosphoproteomics and RTK-centric signalling maps show that TrkB-rich nodes frequently coexist with activated ALK, MET, RET, and IGF-1R, converging on ERK and AKT to maintain *MYCN*-driven transcriptional programs and buffer against pathway-specific inhibitors [39,42]. This redundancy offers a mechanistic basis for intrinsic and acquired resistance to targeted therapies directed at single RTKs. Receptor isoform diversity adds yet another layer of functional versatility. While the full-length isoform TrkB.FL supports canonical kinase-dependent survival pathways, the truncated isoform TrkB.T1 modulates BDNF internalisation, calcium dynamics, cytoskeletal organisation, and long-term transcriptional reprogramming [47]. MES-like NB preferentially express TrkB.T1, which orchestrates survival under metabolic and therapeutic stress through endosomal and Ca^2+^-dependent mechanisms [47]. Endosomal signalling by TrkB.FL and TrkB.T1 contributes to prolonged ERK and AKT activation and to altered cell polarity and migration, indicating that isoform composition and trafficking dynamics must be considered when designing therapeutic strategies targeting *NTRK2* [40,42,47] (Figure 1).

#### 2.2.3. TrkC/*NTRK3*: Neuronal Lineage Maintenance and Context-Dependent Apoptosis

TrkC/*NTRK3*, although historically less studied, plays a pivotal role in maintaining neuronal lineage integrity and favourable clinical behaviour. Early clinical series showed that TRKC expression, alone or alongside TRKA, is enriched in well-differentiated, regressing tumours and associates with favourable outcome, mirroring the pattern observed for TRKA-positive disease [4,5,20]. In contemporary multi-omics datasets, *NTRK3* transcripts cluster with ADRN/NOR signatures and differentiation-associated transcriptional circuits, often overlapping with *NTRK1*-positive subgroups and segregating away from MES-like, TrkB-driven states [7,8,17,39]. At the signalling level, engagement of TrkC by NT-3 activates ERK and PI3K-AKT pathways together with PLCγ1-dependent Ca^2+^ signalling, promoting neurite extension, cytostasis, and neuronal differentiation programs when survival co-signals are intact [3,25]. However, under reduced trophic support or impaired PI3K/AKT signalling, TrkC can behave as a dependence receptor, favouring caspase-dependent apoptosis rather than differentiation, in line with the paradigm described for other neurotrophin receptors and p75NTR [26]. This dual potential provides a mechanistic explanation for why TRKC expression tracks with favourable biology: in supportive, Schwannian-rich or ADRN-biased niches it stabilises differentiation, whereas in suboptimal environments it can promote elimination of inadequately supported tumour cells [4,5,14,16,20,25] (Figure 1).

#### 2.2.4. p75NTR/*NGFR*: A Molecular Switch for Differentiation Versus Apoptosis

The low-affinity neurotrophin receptor p75NTR (*NGFR*) sculpts neurotrophin signalling and NB cell fate decisions by acting both as a context-dependent modulator of Trk output and as an autonomous death receptor [3,26]. Structurally, p75NTR contains a cysteine-rich extracellular domain that binds mature neurotrophins and proneurotrophins, and an intracellular death domain that couples to JNK, NF-κB, and caspase pathways [26].

When co-expressed with TrkA, p75NTR increases affinity and selectivity for NGF, stabilising high-affinity binding sites and biasing signalling towards differentiation and survival, thereby reinforcing the ADRN/NOR phenotype typical of favourable NB [3,4,14,20,26]. In contrast, association with sortilin generates a receptor complex with high affinity for proneurotrophins (proNGF and proBDNF), which preferentially triggers JNK activation, mitochondrial cytochrome-c release, and caspase-dependent apoptosis [26,27]. This dual behaviour turns p75NTR into a conditional switch: in NGF-rich, Schwannian stroma-associated microenvironments, it synergises with TrkA/TrkC to maintain neuronal differentiation and cytostasis, whereas in ligand-poor or stress conditions, or in the presence of proneurotrophins, it promotes apoptotic clearance of tumour cells that cannot be adequately supported [4,14,16,20,26,27].

Collectively, these findings position p75NTR as a central molecular switch within the neurotrophin network. It fine-tunes the affinity and qualitative output of TrkA/TrkC in favourable, ADRN-differentiated states and, when epigenetically reactivated and partnered with sortilin, reinstates an apoptosis-competent program exploitable by proneurotrophins and NGFR-directed immunotoxins [3,4,14,16,20,26,27,48,49,50,51] (Figure 1). A structured comparison of receptor-associated cell states, signalling pathways, and epigenetic regulatory mechanisms is provided in Table 1.

### 2.3. Epigenetic Mechanisms Regulating Trk Receptors and Neurotrophic Signalling in NB

Epigenetic mechanisms are heritable and reversible modifications that regulate gene expression without altering the DNA sequence through chemical changes to DNA and associated proteins [52]. They determine whether genes are activated or silenced according to the biological context [53]. The main mechanisms include DNA methylation, histone modifications, and non-coding RNAs [52,54]. DNA methylation at CpG islands typically promotes gene silencing by inducing chromatin compaction [52,55]. Histone acetylation enhances transcription by relaxing chromatin, while specific histone methylation marks are associated with either activation (H3K4me3) or repression (H3K27me3) [52,55]. Non-coding RNAs, including microRNAs and long non-coding RNAs, further fine-tune gene expression by targeting mRNAs or modulating chromatin structure [52,55].

**Figure 2:**
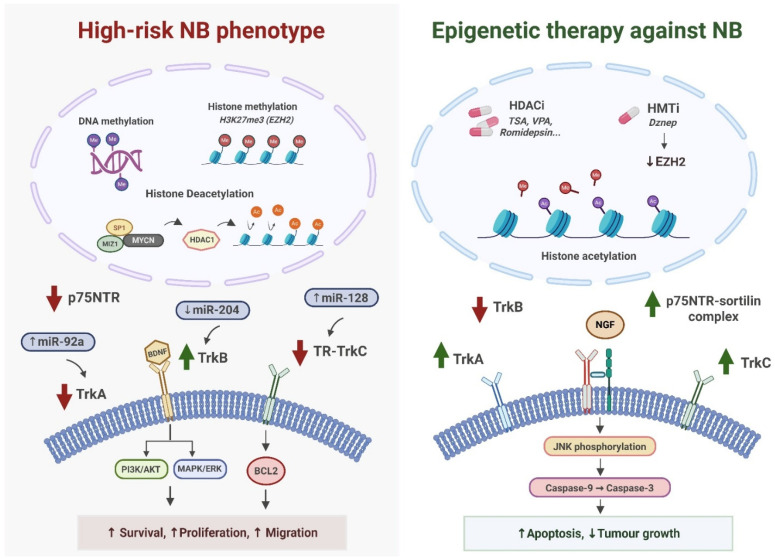
Epigenetic control of neurotrophin receptor signalling in neuroblastoma. Epigenetic mechanisms regulating Trk receptors and neurotrophin signalling in neuroblastoma. In unfavourable high-risk NB (**left**), promoter DNA hypermethylation, PRC2/EZH2-mediated H3K27me3, HDAC1-dependent deacetylation, MYCN-driven repression, and dysregulated miRNAs (miR-92a, miR-204, and miR-128) suppress TrkA, TrkC, and p75NTR while promoting TrkB, leading to activation of PI3K/AKT and MAPK/ERK pathways, increased BCL2 expression, and enhanced survival, proliferation, and migration. Epigenetic therapies (**right**), including HDAC and EZH2 inhibitors, restore histone acetylation and rebalance Trk receptor expression, leading to caspase activation, cell death, and subsequent tumour growth. **Abbreviations:** NB, neuroblastoma; HDACi, histone deacetylase inhibitors; TSA, trichostatin A; VPA, valproic acid; HMTi, histone methyltransferase inhibitors; EZH2, enhancer of zeste homolog 2; DZNep, 3-deazaneplanocin A; Trk, tropomyosin receptor kinase; p75NTR, p75 neurotrophin receptor; NGF, nerve growth factor; BDNF, brain-derived neurotrophic factor; PI3K, phosphoinositide 3-kinase; AKT, protein kinase B; MAPK/ERK, mitogen-activated protein kinase/extracellular signal-regulated kinase; JNK, c-Jun N-terminal kinase; BCL2, B-cell lymphoma 2; miR, microRNA.

These epigenetic mechanisms contribute to the regulation of neurotrophin receptors across multiple cancers, including colorectal carcinoma, ovarian cancer, and NB, where neurotrophin signalling is largely shaped by epigenetic regulation and microenvironmental influences rather than by recurrent fixed genetic alterations [29,32,56,57,58], highlighting the central role of epigenetic plasticity in NB biology and therapeutic vulnerability. The multilayered epigenetic regulation of Trk receptors in favourable versus high-risk NB, integrating DNA methylation, Polycomb repressive complex 2/Enhancer of Zeste homolog 2 (PRC2/EZH2)-dependent chromatin repression, MYCN-driven silencing, and miRNA-mediated control, is schematically summarised in Figure 2.

#### 2.3.1. DNA- and Histone-Based Epigenetic Regulation of Neurotrophin Receptor Expression

The primary epigenetic mechanism regulating neurotrophin receptors and signalling in NB is promoter DNA methylation, which represses gene transcription through the addition of methyl groups to cytosine residues within CpG-rich genomic regions. A study by Lau and colleagues demonstrated that the neurotrophic tyrosine kinase receptors *NTRK1*, *NTRK2*, and *NTRK3* are frequent targets of aberrant hypermethylation, with *NTRK1* and *NTRK3* reported to be hypermethylated in approximately 100% and 90% of primary NB tumour samples, respectively [58]. This was further corroborated by additional findings highlighting the role of both direct DNA methylation and histone methylation mediated by EZH2, a core catalytic component of the PRC2. These epigenetic mechanisms act at the *NTRK1* P1 and P2 promoters, promoting promoter methylation and chromatin repression, ultimately leading to epigenetic silencing of *NTRK1* transcription, particularly in unfavourable NB tumours [30].

The neurotrophin receptor p75NTR (*NGFR*) has been identified as an epigenetic target of EZH2 within the PRC2. In human NB cell lines, including SMS-KCNR, SH-SY5Y, and NGP, enrichment of PRC2 components and the repressive histone marker H3K27me3 at the *NGFR* promoter correlates with transcriptional silencing of *NGFR* and maintenance of an undifferentiated cellular phenotype [29]. Conversely, pharmacological inhibition of EZH2 with 3-deazaneplanocin A (DZNep), as well as RNA-interference-mediated EZH2 knockdown in SMS-KCNR cells, significantly upregulates *NGFR* expression and induces cellular differentiation, as demonstrated by neurite outgrowth [29]. This indicates that EZH2-mediated repression of *NGFR* plays a critical role in maintaining the undifferentiated state of NB cells and highlights this epigenetic axis as a potential therapeutic target to promote tumour cell differentiation.

Although neurotrophin signalling systems are influenced by global epigenetic programs, transcriptional regulation of individual neurotrophin genes also depends on promoter-specific sensitivity to DNA and histone modifications. In a study using mouse NB Neuro-2a cells, treatment with the DNA methyltransferase inhibitor 5-aza-2′-deoxycytidine (5-aza-dC) increased BDNF transcription through CpG demethylation of promoter I, leading to induction of BDNF exon I–IX mRNA transcripts [59]. In addition, treatment with the histone deacetylase (HDAC) inhibitor trichostatin A (TSA) increased acetylation of histones H3 and H4 at *BDNF* promoter I, further supporting the contribution of histone acetylation to BDNF transcriptional regulation [59]. However, this regulatory paradigm is not universally applicable across all neurotrophins. Although the NT-3 promoter IB is also hypermethylated in Neuro-2a cells, NT-3 expression was not induced by either 5-aza-dC or TSA, indicating that DNA methylation and histone acetylation do not regulate *NT-3* transcription in this specific NB model in the same manner as *BDNF* [59]. This emphasises the importance of epigenetic fine-tuning of neurotrophin expression in NB and underscores the existence of gene-specific regulatory mechanisms that shape neurotrophin expression in a context-dependent manner.

In addition to classical epigenetic regulatory mechanisms, the oncogenic transcription factor MYCN can function as a direct transcriptional repressor of neurotrophin receptor genes, including *NTRK1* and *p75NTR* [28]. Studies performed in NB cell lines, such as TET-21/N, SK-N-BE, SH-SY5Y, and LAN-1, as well as in MYCN-overexpressing transgenic mouse models, have demonstrated that MYCN directly targets proximal/core promoter regions of these neurotrophin receptor genes [28]. MYCN forms a transcriptional repression complex with SP1 and MIZ1 and subsequently recruits histone deacetylase 1 (HDAC1), promoting a repressive chromatin configuration that leads to decreased receptor expression [28]. Through downregulation of these receptors, MYCN reduces cellular responsiveness to NGF signalling, allowing tumour cells to evade NGF-mediated apoptosis and promoting progression towards a more malignant phenotype [28]. Importantly, this transcriptional repression can be pharmacologically reversed using HDAC inhibitors, such as TSA, which restore *NTRK1* and *p75NTR* expression and re-sensitise cells to apoptotic signalling.

Given the critical role of epigenetic regulation in NB biology, epigenetic drugs have emerged as promising therapeutic strategies capable of modulating neurotrophin-dependent signalling pathways. HDAC inhibitors, including valproic acid (VPA), entinostat, romidepsin, TSA, and sodium butyrate, have been shown to downregulate TrkB expression at both mRNA and protein levels in retinoic-acid-differentiated human NB cell lines, including SH-SY5Y, LAN-1, and Kelly [60]. This downregulation is associated with inhibition of downstream signalling pathways, including PLCγ1, PI3K/Akt, and ERK/MAPK, which are activated by BDNF [60]. In the case of VPA, this effect has been linked to significant depletion of EZH2, resulting in derepression and upregulation of the transcription factor RUNX3, which acts as a negative regulator of *TrkB* gene expression [60].

Interestingly, in contrast to TrkB downregulation, VPA treatment in RA-differentiated SH-SY5Y cells results in the upregulation of the pro-apoptotic receptors p75NTR and TrkC [48]. This mechanism was further investigated in a subsequent study, which demonstrated that VPA-mediated reduction of EZH2 promotes derepression and upregulation of the transcription factor CASZ1, a positive regulator that binds the *NGFR* promoter and increases NGFR transcription [48]. In parallel, VPA treatment also increases expression of the co-receptor sortilin, leading to increased formation of the p75NTR/sortilin receptor complex at the plasma membrane [48]. This shift in neurotrophin receptor balance increases cellular sensitivity to proNGF stimulation and promotes activation of pro-apoptotic signalling cascades involving phosphorylation of JNK and c-Jun, activation of caspases 9 and 3, and cleavage of PARP [48]. Together with TrkB downregulation, these effects contribute to the antitumoral efficacy of VPA in NB. Similarly, treatment of NB cells with the HDAC inhibitor depsipeptide reduces binding of PRC2 components at the *NGFR* promoter and increases p75NTR expression [29]. In addition, in vivo xenograft studies using nude mice bearing SMS-KCNR tumours have shown that pharmacological inhibition of EZH2 using DZNep significantly reduces tumour volume and promotes morphological differentiation, further supporting the therapeutic potential of targeting epigenetic repression of neurotrophin receptors in NB [29]. As combination therapeutic strategies are increasingly explored in NB, epigenetic upregulation of p75NTR may also be exploited to sensitise NB cells to antibody-based therapeutic approaches, including immunotoxins, thereby improving treatment efficacy [50].

Neurotrophin receptor signalling in NB is, therefore, regulated through the coordinated action of promoter DNA methylation, PRC2/EZH2-mediated histone methylation, and HDAC-dependent chromatin remodelling. These epigenetic processes collectively influence tumour differentiation, survival, and therapeutic responsiveness, highlighting epigenetic modulation as a promising strategy to reprogram malignant NB phenotypes.

#### 2.3.2. miRNA-Mediated Regulation of Neurotrophin Receptors

Aside from DNA- and histone-based epigenetic programs, research indicates that the regulation of neurotrophin receptors is strongly linked to miRNA-dependent mechanisms and is, interestingly, isoform-specific. A study by Guidi and colleagues has shown that the full-length kinase-active isoform (FL-NTRK3) and the non-catalytic truncated isoform (TR-NTRK3) are regulated by different sets of miRNAs in the SH-SY5Y NB cell line [32]. The study has reported that while miR-151-3p represses the full-length receptor, a different set of miRNAs, including miR-128, miR-485-3p, miR-765, and miR-768-5p, targets the truncated version of NTRK3 [32]. Moreover, this epigenetic repression carried out by miR-151-3p on FL-NTRK3 results in the suppression of the Ras/MAPK signalling pathway, known to promote cell survival and proliferation in NB [61]. On the other hand, the overexpression of miR-128 in SH-SY5Y cells leads to a decrease of TR-NTRK3 that is concomitant to morphological changes, such as rounded cell bodies and shorter neurites, along with a significant increase in SH-SY5Y cell number, which has been associated with the upregulation of the anti-apoptotic factor BCL2 [32].

In this context, MYCN-regulated microRNAs have been examined as modulators of post-transcriptional control of TrkA in NB. In particular, the miRNA-17–92 cluster, which includes miR-92a, a microRNA whose expression is elevated in high-risk NB [62], has been investigated in the BE(2)-M17 NB cell line. The overexpression of miR-92a has reduced TrkA mRNA and protein levels, resulting in enhanced tumour cell proliferation and migration, whereas inhibition of miR-92a produced the opposite effect and was accompanied by suppression of tumour cell growth [63]. These findings establish a post-transcriptional mechanism through which elevated miR-92a can functionally silence the tumour-suppressive activity of TrkA. In addition, a set of 37 microRNAs has been identified as correlating with TrkA expression [13]. Within this group, miR-542-5p emerged as the most strongly TrkA-associated microRNA and was inversely linked to MYCN amplification and poor survival, while effectively distinguishing between localised and metastatic disease [13].

The interplay between distinct epigenetic mechanisms has been highlighted in the regulation of Trk receptors in NB. One primary mechanism involves hypermethylation of the thyroid transcription factor-1 (TTF1) promoter and its interaction with microRNA-dependent pathways [64]. As TTF1 is a key transcription factor in lung and thyroid development [65], its expression in NB patient tissues has been examined in relation to TrkA and TrkB levels [64]. Transcriptional repression of TTF1, driven by promoter hypermethylation, was associated with decreased TrkA expression in both differentiated and undifferentiated NB tissues compared with highly differentiated benign tumours, referred to as ganglioneuromas (GNs) [64].

In contrast to TrkA, TrkB protein levels were elevated in both differentiated and undifferentiated NB relative to GN [64]. Notably, miR-204, a transcriptional target of TTF1, was also downregulated in these tumours, and a positive correlation was observed between miR-204 and TTF1 mRNA expression [64]. As TrkB is a direct target of miR-204, its downregulation led to increased TrkB expression, which is commonly associated with high-risk NB and promotes tumour cell survival, migration, and invasion. This role of TTF1 promoter methylation in modulating the TrkA/TrkB balance through miR-204 was further validated across multiple NB cell lines, including SK-N-BE, SH-SY5Y, SK-N-SH, and IMR-32 [64]. The functional relevance of this epigenetic–neurotrophin interaction was confirmed in vivo using a NB xenograft model generated from SK-N-BE cells. Tumours derived from TTF1-overexpressing cells exhibited reduced growth, increased TrkA expression, decreased TrkB levels, and enhanced expression of neurogenic differentiation markers, thereby reinforcing the role of neurotrophin receptor regulation in shaping NB tumour behaviour [64].

**Table 1:** Neurotrophin receptors in neuroblastoma. A summary of the major neurotrophin receptors implicated in NB, their cell-state associations, downstream signalling pathways, functional roles, and regulatory mechanisms.

Receptor	Expression/Cell-State Association	Main Pathways	Functional Consequences	Epigenetic/Regulatory Mechanisms	References
TrkA/NTRK1	Favourable NB; ADRN/NOR identity; super-enhancer circuitry; spontaneous regression	ERK1/2, PI3K-AKT, PLCγ1–Ca^2+^; JNK/p38 (NGF deprivation)	Differentiation, neurite outgrowth, cytostasis; dependence-receptor apoptosis	Promoter hypomethylation; MYCN–SP1–MIZ1 repression; PRC2/EZH2 silencing	[1,2,3,4,5,8,14,17,20,25,28,29,30,45]
TrkB/NTRK2 (FL)	MES-like, aggressive, therapy-resistant NB; BDNF autocrine loop	AKT–mTORC1, ERK1/2, STAT3, NF-κB	Proliferation, angiogenesis, metabolic plasticity; MES transition	BRD4 super-enhancers; IL-6/STAT3 reinforcement; enhancer rewiring	[6,7,8,9,21,24,34,39,41,66]
TrkB.T1	Predominant in MES states and hypoxic niches	Endosomal Ca^2+^ signalling; cytoskeletal remodelling	Stress survival, invasion, plasticity	Isoform-specific regulation; MES reinforcement	[13,17,40,42,47]
TrkC/NTRK3	Favourable ADRN phenotype; clusters with NTRK1	ERK, PI3K-AKT, PLCγ1–Ca^2+^	Differentiation; dependence-receptor apoptosis	PRC2/EZH2 repression; miR-128 targeting	[4,5,17,25,26,29,30,32,49]
p75NTR/NGFR	High in favourable NB; reduced in MYCN-amplified tumours	JNK, NF-κB, caspases; proneurotrophin–sortilin axis	Differentiation vs apoptosis switch	MYCN repression; EZH2 silencing; HDAC reactivation; VPA induction	[3,26,27,28,29,48,49,50,51]

**Abbreviations:** NB, neuroblastoma; Trk, tropomyosin receptor kinase; NTRK, neurotrophic receptor tyrosine kinase; FL, full-length; TrkB.T1, truncated isoform of TrkB; ADRN, adrenergic; NOR, noradrenergic; MES, mesenchymal; NGF, nerve growth factor; BDNF, brain-derived neurotrophic factor; ERK1/2, extracellular signal-regulated kinases 1 and 2; PI3K, phosphoinositide 3-kinase; AKT, protein kinase B; PLCγ1, phospholipase C gamma 1; Ca^2+^, calcium ion; JNK, c-Jun N-terminal kinase; p38, p38 mitogen-activated protein kinase; mTORC1, mechanistic target of rapamycin complex 1; STAT3, signal transducer and activator of transcription 3; NF-κB, nuclear factor kappa B; PRC2, polycomb repressive complex 2; EZH2, enhancer of zeste homolog 2; SP1, specificity protein 1; MIZ1, Myc-interacting zinc finger protein 1; BRD4, bromodomain-containing protein 4; IL-6, interleukin 6; miR, microRNA; p75NTR, p75 neurotrophin receptor; NGFR, nerve growth factor receptor; HDAC, histone deacetylase; VPA, valproic acid.

### 2.4. Microenvironmental Reinforcement, Trafficking Dynamics, and Therapeutic Implications

Beyond transcriptional and epigenetic regulation, neurotrophin signalling in NB is profoundly shaped by receptor trafficking and spatial compartmentalisation. After ligand engagement, Trk receptors internalise via clathrin-dependent endocytosis and continue to signal from Rab5- and Rab11-positive endosomal compartments, where ERK and AKT activation can be sustained independently of surface inputs [3,39,47]. In NB, this endosomal signalling is not a simple extension of plasma-membrane activity: altered vesicular flux, particularly in TrkB-high and MES-like states, prolongs phosphorylation events, enhances survival under oxidative or metabolic stress, and facilitates invasive behaviour in hypoxic or nutrient-deprived niches [39,41,42,46,47].

These spatial mechanisms intersect with microenvironmental pressures that remodel neurotrophin pathways in discrete tumour regions. Spatial transcriptomics has shown that TrkB-high NB nests are surrounded by IL-6- and TNF-α-producing macrophages, fibroblasts secreting ECM components that promote MES conversion, and endothelial cells releasing BDNF-containing vesicles [9]. These stromal cues converge on STAT3, NF-κB, and HIF-1α, stabilising *NTRK2* transcription and reinforcing TrkB-driven malignancy, thereby creating protected signalling niches resistant to chemotherapy and targeted agents [21,32,41,46,66]. Notably, these cytokine-activated pathways do not merely sustain transcriptional outputs but actively reprogram the epigenetic landscape of NB cells. Inflammatory cytokines, such as IL-6 and TNF-α, abundantly produced within the tumour microenvironment, activate STAT3 and NF-κB signalling cascades that directly interface with chromatin-modifying machinery. STAT3 has been shown to recruit histone acetyltransferases and to cooperate with polycomb repressive complexes, while NF-κB signalling promotes dynamic changes in histone acetylation and enhancer activation, collectively reshaping chromatin accessibility and transcriptional plasticity [67,68]. In NB, these epigenetic alterations are tightly linked to metabolic rewiring. Cytokine-driven signalling enhances glycolytic flux, promotes mitochondrial adaptability, and increases resistance to oxidative stress, thereby enabling tumour cells to survive under hypoxic and nutrient-deprived conditions typical of aggressive niches [9,69]. Importantly, these metabolic adaptations feed back into chromatin regulation by influencing metabolite availability (e.g., acetyl-CoA and NAD^+^), further stabilising epigenetic states associated with mesenchymal transition and therapy resistance. Within this framework, TrkB signalling integrates with inflammatory and metabolic cues to reinforce pro-survival programs. IL-6/STAT3 and NF-κB activation sustain *NTRK2* expression and downstream PI3K-AKT and MAPK pathways, while simultaneously promoting MYC-family oncogenic activity, establishing a feed-forward loop that links inflammatory signalling, epigenetic plasticity, and metabolic adaptation. This coordinated axis ultimately sustains TrkB-driven tumour aggressiveness, metastatic potential, and resistance to therapy [70].

Macrophage–NB crosstalk further drives MES identity via IL-6/STAT3 and c-MYC upregulation, providing a direct mechanistic link between inflammatory stroma, MYC-family oncogene activation, and *NTRK2*-dependent resistance [39,66,71].

In contrast, TrkA/TrkC-positive ADRN-like regions preferentially localise to Schwannian-rich stroma and vascular domains enriched in NGF and NT-3, which support differentiation, cytostasis, and apoptotic priming [4,14,17,20]. This spatial heterogeneity generates coexisting tumour “ecosystems” in which neurotrophin signalling is continuously recalibrated by ligand availability, cytokine gradients, ECM organisation, and oxygen tension [9,11,17,39].

Advanced preclinical models increasingly capture these microenvironmental dependencies more faithfully than classical monolayer cultures (Table 2). Three-dimensional NB spheroids recreate neurotrophin gradients, hypoxic cores, and ECM interactions that reshape Trk signalling and alter sensitivity to HDAC, ALK, and TRK inhibitors compared with 2D systems [72]. Collagen-based scaffolds and biomimetic matrices demonstrate that ECM stiffness and architecture modulate TrkB phosphorylation, cytoskeletal dynamics, and treatment responses [73]. Bone-mimetic 3D-printed models reveal that osteoblast- and osteoclast-like cells modulate TrkB-driven programs and contribute to bone metastatic tropism [74].

**Table 2:** Advanced preclinical models in neuroblastoma. Advanced three-dimensional and co-culture model systems are used to study NB, emphasising their biological context, dominant signalling features, and translational applications.

Model System	Biological Context	Key Signalling Features	Applications	References
NB 3D spheroids	Oxygen/nutrient gradients; ADRN/MES coexistence	Hypoxia → HIF-1α → TrkB upregulation	TRK/ALK inhibitor testing; epigenetic screens	[9,41,46,72]
Collagen scaffolds	ECM-driven MES identity	Stiffness-dependent TrkB activation	Invasion and therapy modelling	[73]
3D bone-mimetic systems	Osseous microenvironment	STAT3/c-MYC activation; NTRK2 reinforcement	Bone metastasis modelling	[74]
Patient-derived organoids	Preserve genetic and epigenetic heterogeneity	Enhancer switching; regional Trk expression	Precision therapy testing	[75,76,77,78]
Organoid–stroma co-culture	NB + fibroblasts/endothelium	BDNF secretion; MES reinforcement	Niche-driven MES modelling	[9,75,76,77,78]
Organoid–immune co-culture	TAM/T-cell interaction	IL-6/STAT3 → NTRK2 upregulation	Immunotherapy evaluation	[66,71,78]

**Abbreviations:** NB, neuroblastoma; ADRN, adrenergic; MES, mesenchymal; ECM, extracellular matrix; HIF-1α, hypoxia-inducible factor 1 alpha; Trk, tropomyosin receptor kinase; ALK, anaplastic lymphoma kinase; STAT3, signal transducer and activator of transcription 3; c-MYC, MYC proto-oncogene, bHLH transcription factor; NTRK2, neurotrophic receptor tyrosine kinase 2; BDNF, brain-derived neurotrophic factor; TAM, tumor-associated macrophage; IL-6, interleukin 6.

Patient-derived NB organoids (PDOs) and organoid–stroma cocultures now recapitulate tumour heterogeneity, macrophage-driven MES induction, and niche-specific survival pathways, providing platforms to evaluate rational combinatorial therapies under physiologically relevant conditions [75,76,77,78]. Recent PDO systems integrating fibroblasts, endothelial cells, and tumour-associated macrophages faithfully reproduce in vivo metabolic, chromatin, and receptor-state heterogeneity, demonstrating that *NTRK2* upregulation and MES transition require continuous stromal reinforcement and cannot be stably maintained in tumour cells in isolation [39,75,76,77,78].

Although canonical *NTRK* gene fusions are rare in NB, the remarkable clinical efficacy of TRK inhibitors in fusion-positive paediatric solid tumours has firmly established the neurotrophin signalling axis as a therapeutically actionable target, with important implications for NB [3,35,79,80]. In fusion-driven malignancies, Trk receptors are constitutively active and ligand-independent, resulting in persistent activation of downstream signalling cascades, including RAS/MAPK, PI3K-AKT, and PLCγ pathways, which promote uncontrolled proliferation and survival [81,82,83,84]. These tumours typically exhibit high sensitivity to first-generation TRK inhibitors, such as larotrectinib and entrectinib; however, resistance frequently arises through on-target kinase domain mutations, such as solvent-front, gatekeeper, and xDFG substitutions, or through reactivation of downstream signalling pathways, particularly MAPK/ERK, as well as compensatory activation of parallel RTKs [35,78,79,85,86,87].

In contrast, *NTRK* dysregulation in NB is predominantly epigenetic rather than genomic, and Trk signalling remains tightly dependent on ligand availability [5]. Full-length TrkA and TrkC receptors require binding to NGF or NT-3, respectively, and can function as dependence receptors, triggering apoptosis via p75NTR and JNK pathways in the absence of ligands. This mechanism contributes to the favourable prognosis and spontaneous regression often observed in TrkA or TrkC-high NB [84,88]. Conversely, TrkB expression is associated with aggressive disease, frequently sustained by an autocrine BDNF loop, particularly in MYCN-amplified tumours, leading to chronic activation of PI3K-AKT and MAPK signalling pathways that enhance tumour survival, angiogenesis, and chemoresistance [5,89].

Despite the potential of TRK inhibition to disrupt these oncogenic programs, its efficacy in NB is often limited by adaptive resistance mechanisms. These include the activation of alternative RTKs, such as ALK, MET, RET, IGF-1R, and AXL, as well as downstream signalling convergence on RAS-MAPK and PI3K-AKT pathways [39,41,42]. Furthermore, intratumoral heterogeneity plays a critical role in therapeutic resistance, as spatially distinct subclonal populations, ranging from ADRN-like, TrkA/TrkC-positive regions to TrkB-high MES clusters, can be differentially selected under TRK blockade, enabling the emergence of resistant cellular compartments [2,6,7,8,9,17,23,24,39].

In this context, modulating neurotrophin receptor expression through epigenetic priming may represent a promising strategy to limit tumour cell plasticity and attenuate compensatory RTK signalling. Restoring TrkA and TrkC expressions could promote a more differentiated and less adaptable tumour phenotype [90,91]. However, this warrants further investigation to fully define its therapeutic potential in NB.

Overall, neurotrophin signalling in NB emerges from the integration of receptor trafficking, microenvironmental forces, niche-derived cytokines, ECM architecture, and epigenetic configuration. These layers orchestrate the balance between TrkA/TrkC-driven differentiation and TrkB-mediated MES adaptation, shaping metastatic proclivity, therapeutic response, and the potential for tumour reprogramming [2,6,7,8,9,11,12,13,18,23,39,79] (Table 3). This complexity strongly supports therapeutic strategies that combine TRK inhibition with epigenetic modulators, ALK/MEK blockade, or differentiation agents, evaluated specifically in 3D and organoid platforms capable of preserving in vivo heterogeneity and tumour–stroma reciprocity [34,39,76,77,78,79,82,83,84,85].

## 3. Epigenetic Plasticity, Clonal Diversity, and Temporal Dynamics in NB

### 3.1. Epigenetic Plasticity as a Driver of Intratumoral Heterogeneity

Reminiscent of neural crest progenitors, with epigenetic priming enabling rapid adaptation to microenvironmental cues and therapeutic stress, NB is characterised by pronounced epigenetic plasticity, which underlies its remarkable intratumoral heterogeneity [92] and ability for dynamic cell-state transitions [13,93]. Together, these features define NB as a developmentally encoded, epigenetically driven malignancy, in which non-genetic mechanisms contribute substantially to tumour evolution, therapy response, and disease progression. Lundberg et al. provide a comprehensive overview of how advances in molecular profiling, particularly at single-cell and epigenomic levels, are reshaping our understanding of NB biology and opening new therapeutic avenues. The review summarises how single-cell technologies, including single-cell epigenomics and transcriptomics, have unveiled diverse malignant subpopulations and developmental trajectories within tumours that were previously unknown [94].

### 3.2. Epigenomic Regulation of ADRN–MES States and Trk-Associated Identities

The evolution of NB is shaped by reversible epigenomic programs that allow cells to shift between distinct transcriptional identities. The coexistence of ADRN and MES states within individual tumours, together with intermediate and hybrid populations, has been demonstrated [95], and these states appear to be maintained and interconverted through dynamic regulation of chromatin accessibility, histone modifications, and DNA methylation.

Large-scale DNA methylation profiling has reinforced the central role of epigenetic heterogeneity in NB by identifying subtype-specific and risk-associated methylation patterns while also revealing substantial variability within tumours [96]. By analysing DNA methylation profiles from over 300 NB cases, tumours could be assigned to methylation-defined classes, such as “MYCN type”, telomere maintenance mechanism (TMM)-positive, and TMM-negative, each associated with distinct clinical outcomes, supporting the relevance of epigenetic states for NB classification and prognosis.

### 3.3. Temporal Remodelling of Trk Signalling Networks

A prominent axis within this heterogeneity is the differential and temporally regulated expression of neurotrophic Trk receptors. As previously mentioned, TrkA is strongly associated with favourable outcome, whereas TrkB frequently co-expressed with BDNF characterises aggressive NB subpopulations with stem-like and MES features, enhanced survival signalling, and resistance to cytotoxic and targeted therapies [97]. These Trk-associated programs align with ADRN and MES transcriptional identities, linking developmental specification to epigenetically controlled survival and resistance pathways. What is important to note is that Trk receptor signalling is not static but undergoes temporal remodelling. Proteomic and phosphoproteomic analyses indicate that Trk-dependent signalling networks are extensively reorganised following ligand stimulation or therapeutic perturbation, including changes in protein–protein interactions, post-translational modifications, and downstream pathway engagement [98]. These observations support the notion that NB cells dynamically reprogram gene regulation and signalling to adapt to stress and facilitate state transitions.

Single-cell epigenomic approaches, including scATAC-seq, single-cell DNA methylome profiling, and spatially resolved multi-omics, now enable the identification of cell-state-specific regulatory elements and chromatin accessibility programs that stabilise or promote transitions between Trk-defined phenotypes. These technologies provide opportunities to therapeutically target epigenetic circuits maintaining NB plasticity, rather than single static cell states [99].

### 3.4. Epigenetic Therapies as Modulators of Plasticity and Adaptive Resistance

Preclinical studies suggest that inhibition of epigenetic modifiers can restrain adaptive transcriptional programs, stabilise differentiation states, and sensitise NB cells to chemotherapy or targeted agents. While epigenetic plasticity enables resistance, its reversibility may expose transient vulnerabilities exploitable through rational combinations and temporally optimised treatment schedules.

Jubierre et al. demonstrated that BET inhibitors, such as JQ1 or OTX015, reduce MYCN expression, decreasing cell growth and enhancing therapeutic impact, with synergistic effects observed when combined with HDAC inhibitors [34,100]. Jiménez et al. showed that HDAC inhibitors, such as entinostat, remodel epigenetic landscapes and increase expression of immune-related receptors, potentially sensitising NB cells to immunotherapy [101]. Endo et al. further reported that EZH2 inhibition induces differentiation-associated gene expression and suppresses proliferation, and that combined EZH2 and DNMT inhibition potentiates these effects even in resistant cells, consistent with derepression of differentiation programs and suppression of MYCN/c-MYC oncogenic circuitry [102].

### 3.5. Clonal Evolution, Genetic and Non-Genetic Heterogeneity, and Therapy-Driven State Transitions

Within the malleable epigenetic landscape of NB, intratumoral heterogeneity manifests as clonal diversity that is not exclusively genetically determined, but instead arises from the interplay between genetic alterations, epigenetic regulation, and transcriptional plasticity. Longitudinal and time-resolved studies indicate that NB does not evolve through linear clonal succession; rather, tumour progression reflects dynamic remodelling of transcriptional and epigenetic states within genetically related populations.

Using paired tumour biopsies and longitudinal circulating cell-free DNA (cfDNA) analysis it has been demonstrated that relapse is most frequently driven by the selective expansion of pre-existing minor subclones—often already detectable at diagnosis—rather than by the acquisition of de novo resistance mutations, with treatment selecting for clones enriched in proliferative and survival pathways, particularly RAS/MAPK signalling [103]. Similarly, another study showed that early branching of subclones generates spatially distinct tumour regions and shapes therapeutic response, with resistant populations frequently associated with MES-like features and reactivation of early developmental programs [104]. Additional cohort studies have highlighted distinct temporal trajectories of NB tumorigenesis involving *MYCN* amplification, *ATRX* mutations, telomere maintenance mechanisms, and recurrent structural variants at loci such as *MYCN*, *TERT*, *ATRX*, and *MDM2-CDK4* [105], underscoring the complexity of genetic evolution in high-risk disease [106].

An important contributor to this complexity is extrachromosomal DNA (ecDNA), which introduces substantial intercellular variability in oncogene copy number. Single-cell DNA and RNA sequencing studies have revealed pronounced ecDNA heterogeneity for oncogenes, such as MYCN, directly influencing transcriptional output and downstream gene regulatory programs. This uneven segregation of ecDNA during cell division promotes rapid adaptability of tumour cell states and provides a powerful mechanism for therapy resistance without requiring new genomic mutations [107,108].

Beyond genetic diversity, single-cell analyses demonstrate that non-genetic drivers, including epigenetic variation, transcriptional plasticity, and microenvironmental influences, play a major role in shaping clonal behaviour. These heritable yet reversible states influence proliferation, differentiation, and drug responsiveness independently of DNA sequence changes, enabling tumour cells to adopt stress-tolerant, stem-like, or slow-cycling phenotypes under therapeutic pressure.

In this context, Avitabile et al. used scRNA-seq to show that chemoresistant NB cell populations display distinct transcriptomic programs involving DNA repair, stress responses, and ribosomal and histone metabolism, together with biased lineage shifts consistent with ADRN-to-MES transitions. Importantly, these resistant transcriptional states mirrored intrinsic subpopulations present in patient tumours and correlated with clinical outcome [109]. Complementing these findings, Yu et al. performed longitudinal single-cell multi-omics profiling of matched pre- and post-induction chemotherapy samples from high-risk NB patients, revealing therapy-induced remodelling of both neoplastic and stromal compartments, expansion of MES-like tumour cells, and paracrine signalling mechanisms, such as the HB-EGF–ERBB4 axis, that promote ERK activation and contribute to adaptive resistance [110].

Integrated single-cell transcriptomic, epigenomic, and spatial analyses have further identified intermediate “bridge” states between ADRN and MES programs that are associated with aggressive disease and poor prognosis. These states are supported by latent epigenetic priming and reinforced by microenvironmental cues, highlighting gene regulatory networks and transcription factors that enable rapid cell-state transitions and represent potential targets for therapeutic intervention aimed at limiting maladaptive plasticity [111].

### 3.6. Concluding Remarks: Temporal Adaptation and Therapeutic Implications

NB should no longer be viewed as a static aggregation of genetically defined subtypes, but rather as a temporally adaptive system in which epigenetic configuration, lineage identity, and signalling network topology are continuously reshaped. Within this dynamic landscape, neurotrophin receptors function not merely as prognostic markers but as state-defining nodes that integrate chromatin architecture, oncogenic drivers, metabolic stress, and microenvironmental cues. The balance between TrkA/TrkC-driven differentiation competence and TrkB-mediated mesenchymal adaptation exemplifies how developmental signalling pathways are redeployed to sustain tumour survival. These programs are neither genetically fixed nor uniformly expressed across tumour compartments. Instead, they emerge from reversible enhancer landscapes, MYCN-dependent repression circuits, microRNA networks, and stromal reinforcement, collectively enabling rapid transitions between therapy-sensitive and therapy-tolerant phenotypes. Importantly, this plasticity represents both a challenge and an opportunity. While it underlies adaptive resistance and relapse, it also exposes transient vulnerabilities that may be therapeutically exploitable. Interventions aimed at stabilising adrenergic differentiation states, disrupting BRD4- or EZH2-driven chromatin programs, attenuating IL-6/STAT3-mediated mesenchymal reinforcement, or strategically combining TRK, ALK, and epigenetic inhibitors could limit the cyclic re-emergence of aggressive subclones. Such approaches require temporally informed treatment sequencing and validation in advanced 3D and organoid systems capable of preserving tumour–stroma reciprocity. Future efforts should move beyond single-target paradigms and instead embrace state-oriented therapeutic strategies designed to constrain epigenetic adaptability and collapse malignant signalling flexibility. In this perspective, the neurotrophin–Trk axis represents a conceptual and translational bridge between developmental neurobiology and precision oncology, offering a framework to reinterpret tumour evolution not only as genetic diversification but as controlled plasticity that can, in principle, be redirected.

## Figures and Tables

**Table 3:** Epigenetic therapies targeting Trk-associated axes. Major epigenetic drug classes investigated in NB, focusing on how they modulate Trk receptor-associated signalling networks and influence tumour cell-state plasticity.

Drug Class	Core Mechanism	Effect on Trk Axis	Preclinical Outcome	References
EZH2 inhibitors	PRC2 inhibition → ↓ H3K27me3	Reactivation of *NTRK1/NGFR*. ADRN restoration	Differentiation induction; tumour suppression	[29,30,92]
DNMT inhibitors	Promoter demethylation	Demethylation of *NTRK1/NGFR* loci	Differentiation marker restoration	[18,45,59]
HDAC inhibitors	Chromatin relaxation	↓ TrkB; ↑ p75NTR and TrkC	Apoptosis sensitisation; MES attenuation	[28,36,48,49]
BET inhibitors (JQ1)	Super-enhancer disruption	↓ *NTRK2* and *MYCN*; MES collapse	Synergy with ALK inhibition	[6,34,90]
EZH2 + DNMT combination	Dual chromatin remodelling	Reversal of *MYCN*-driven repression	Synthetic lethality in high-risk NB	[92]

**Abbreviations:** PRC2, polycomb repressive complex 2; H3K27me3, trimethylation of histone H3 at lysine 27; Trk, tropomyosin receptor kinase; NTRK1/2, neurotrophic receptor tyrosine kinase 1/2; NGFR, nerve growth factor receptor; ADRN, adrenergic; DNMT, DNA methyltransferase; HDAC, histone deacetylase; BET, bromodomain and extraterminal domain; JQ1, thienotriazolodiazepine BET inhibitor; ALK, anaplastic lymphoma kinase; MES, mesenchymal.

## Data Availability

No new data were created or analysed in this study.

## Glossary

ADRN	Adrenergic cell state in neuroblastoma, characterised by sympathetic neuron-like features.
AKT	Also known as Protein Kinase B or PKB, is a family of serine/threonine protein kinases critical in regulating cell survival, growth, proliferation, and metabolism.
ALK	Anaplastic lymphoma kinase—a receptor tyrosine kinase involved in cell growth and differentiation, frequently mutated in neuroblastoma.
ATAC-seq	Assay for transposase-accessible chromatin with high-throughput sequencing. A sensitive, low-input method used to map genome-wide chromatin accessibility.
ATRX	A nuclear chromatin remodeller and transcriptional regulator, essential for telomere stability, heterochromatin formation, and histone variant H3.3 deposition.
AXL	Tyrosine kinase receptor, implicated in cell survival, proliferation, and metastasis.
BAD	Bcl-2-associated agonist of cell death, is a pro-apoptotic member of the Bcl-2 family.
BCL2	Anti-apoptotic protein that maintains mitochondrial membrane integrity.
BCL-xL	Anti-apoptotic BCL2 family member, similar in function to BCL2.
BDNF	Brain-derived neurotrophic factor—promotes neuronal survival and differentiation.
BET	Bromodomain and extra-terminal motif proteins, e.g., BRD4—regulators of transcription.
BIM	BCL2-interacting mediator of cell death—a pro-apoptotic protein of the BCL2 family that promotes programmed cell death by neutralising anti-apoptotic proteins like BCL2 and BCL-xL.
BRD4	Bromodomain-containing protein 4—a transcriptional regulator often targeted by BET inhibitors.
CAR-T	Chimeric antigen receptor T-cells—engineered T-cells for targeted cancer therapy.
CASZ1	Zinc finger protein castor homolog 1—a transcription factor involved in neural differentiation.
cfDNA	Cell-free DNA—DNA fragments circulating in blood and other body fluids, often used as a biomarker in cancer.
c-jun	Component of the activator protein-1 (AP-1) proto-oncogene and transcription factor, regulating proliferation, apoptosis, and differentiation.
CpG	Cytosine-phosphate-guanine dinucleotide—DNA sites often involved in methylation and gene regulation.
CREB	cAMP response element-binding protein—a transcription factor that mediates gene expression in response to signals.
DLK1	Delta-like 1 homolog—a protein involved in differentiation and development.
DNMT	DNA methyltransferases—enzymes that add methyl groups to DNA, regulating gene expression.
ELK1	ETS domain-containing protein Elk-1—a transcription factor that regulates gene expression involved in cell proliferation, apoptosis, differentiation, and neuronal function.
ETS	Erythroblast transformation specific—one of the largest families of transcription factors.
ERBB4	Erb-b2 receptor tyrosine kinase 4, also known as HER4, is a member of the epidermal growth factor receptor (EGFR) family, involved in neurodevelopment and cancer.
ERK1/2	Extracellular signal-regulated kinases—central MAPK pathway kinases mediating proliferation and survival.
ecDNA	Extrachromosomal DNA. It is a circular DNA found outside chromosomes, often carrying oncogenes, acting as a key driver of tumour progression.
EZH2	Enhancer of zeste homolog 2—histone methyltransferase enzyme.
FAK	Focal adhesion kinase—mediates integrin signalling and cell migration.
FOXO	Forkhead box O transcription factors—a group of transcription factors that function as master regulators of longevity, stress tolerance, and cellular homeostasis. They regulate apoptosis, cell cycle, and oxidative stress resistance.
FRS2	Fibroblast growth factor receptor substrate 2 is an intracellular adaptor protein that plays a central role in signalling downstream of tyrosine kinases receptors.
GN	Ganglioneuromas—benign tumours of sympathetic nervous tissue.
GATA3	GATA Binding Protein 3—transcription factor critical for sympathetic neuron development.
H3K27me3	Tri-methylation of histone H3 lysine 27—a repressive epigenetic mark.
H3K4me3	Tri-methylation of histone H3 lysine 4—an active transcription mark.
HAND2	Transcription factor involved in neural crest development.
HDAC	Histone deacetylase—removes acetyl groups from histones, silencing gene expression.
HIF-1α	Hypoxia-inducible factor 1-alpha—transcription factor regulating response to low oxygen.
IGF-1R	Insulin-like growth factor 1 receptor—promotes growth and survival.
IL-6	Interleukin-6—cytokine involved in inflammation and tumour progression.
JNK	c-Jun N-terminal kinase—MAPK pathway involved in stress responses.
MCL-1	Myeloid cell leukaemia 1—anti-apoptotic BCL2 family protein.
MDM2-CDK4	Oncogenic co-amplification often seen in cancer.
MES	Mesenchymal cell state in neuroblastoma, associated with stem-like features.
MET	Receptor tyrosine kinase for hepatocyte growth factor—promotes proliferation and motility.
miR-204	MicroRNA involved in TrkA signalling and tumour suppression.
miR-542-5p	MicroRNA inversely correlated with MYCN amplification in neuroblastoma.
miR-92a	MicroRNA elevated in high-risk neuroblastoma.
mTOR	Mechanistic target of rapamycin. It is a crucial protein kinase that functions as a cellular master regulator, controlling cell growth, metabolism, proliferation, and survival based on nutrient availability. It exists in two complexes (mTORC1 and mTORC2), and its hyperactivation is linked to aging and cancer, while its inhibition stimulates autophagy.
MYCN	Oncogenic transcription factor amplified in aggressive neuroblastoma.
NB	Neuroblastoma, a rare aggressive paediatric cancer of the sympathetic nervous system, affecting mostly infants and young children, usually under the age of 5.
NT-3	Neurotrophin-3—growth factor supporting neuron survival and differentiation.
NGF	Nerve growth factor—promotes survival and differentiation of sympathetic neurons.
p21Cip1	Cyclin-dependent kinase inhibitor 1A—regulates cell cycle progression.
p27Kip	Also known as p27, it is a cyclin-dependent kinase inhibitor that controls the development of the cell cycle, namely, stopping cell division in the G0/G1 phase, acting as a tumour suppressor.
p38	Mitogen-activated protein (MAP) kinase responsive to stress and inflammatory signals.
p75NTR (NGFR)	Low-affinity neurotrophin receptor—regulates neuronal survival and apoptosis.
PARP	Poly (ADP-ribose) polymerase—involved in DNA repair.
PDOs	Patient-derived NB organoids—3D in vitro models derived from patient tumours.
PHOX2B	Paired-Like Homeobox 2B is a master regulator gene crucial for the development of the autonomic nervous system and respiratory control.
PI3K	Phosphatidylinositol 3-kinase—a class of enzymes known as lipid kinases, essential intracellular messengers that control cell growth, proliferation, survival, and metabolism.
PI3K-AKT	Signalling pathway promoting growth, survival, and metabolism.
PI3K-AKT-mTOR	Extended signalling cascade integrating growth and nutrient signals.
PLCγ1	Phospholipase C gamma 1—mediates Ca^2+^ signalling downstream of tyrosine kinase receptors.
PLCγ1–IP_3_–Ca^2+^/PKC, JAK/STAT3	Downstream signalling pathways activated by PLCγ1.
PRC2/EZH2	Polycomb repressive complex 2, with EZH2 as the catalytic subunit.
ProBDNF	Precursor of BDNF—can induce apoptosis through p75NTR.
ProNGF	Precursor of NGF—may promote apoptosis via p75NTR.
Rab5- and Rab11-	Positive endosomal compartments—intracellular vesicles involved in trafficking and signalling.
Ras-RAF-MEK-ERK	Canonical MAPK signalling pathway regulating proliferation.
RET	Receptor tyrosine kinase involved in neural development and cancer.
Rho	Family of GTPases regulating cytoskeleton and cell motility.
RTKs	Tyrosine kinase receptors—cell surface receptors activating intracellular signalling.
scATAC-seq	Single-cell ATAC-seq—maps chromatin accessibility at single-cell resolution.
SHC	Adaptor protein linking RTKs to downstream pathways.
Sortilin	A multifunctional type I transmembrane receptor, it drives protein trafficking between the Golgi apparatus, cell surface, and lysosomes.
TMM	Telomere maintenance mechanism—cellular processes that maintain telomere length for continuous division.
TERT	Telomerase reverse transcriptase—catalytic component of telomerase.
TTF1	Thyroid transcription factor-1—transcription factor involved in lung and thyroid development.
TNF-α	Tumour necrosis factor-alpha—cytokine involved in inflammation and apoptosis.
TrkA (NTRK1)	Neurotrophin receptor for NGF—promotes survival and differentiation.
TrkB (NTRK2)	Receptor for BDNF—regulates neuronal survival and plasticity.
TrkC (NTRK3)	Receptor for NT-3—involved in neural differentiation.
VEGF	Vascular endothelial growth factor—stimulates angiogenesis.
**Compounds**	
5-aza-dC	5-aza-2′-deoxycytidine—a nucleoside analogue that inhibits DNA methyltransferases (DNMTs), leading to DNA hypomethylation and reactivation of silenced genes. Commonly used in epigenetic studies and cancer research.
DZNep	3-deazaneplanocin A. It is an inhibitor of S-adenosylhomocysteine hydrolase that indirectly suppresses histone methylation, particularly by targeting the PRC2 complex and reducing H3K27me3 levels.
JQ1	A small-molecule inhibitor of BET (bromodomain and extraterminal domain) proteins that blocks their interaction with acetylated histones, thereby regulating gene transcription.
OTX015	A BET inhibitor similar to JQ1, targeting bromodomain-containing proteins to modulate transcription, with applications in oncology and epigenetic therapy.
TSA	Trichostatin A—a potent histone deacetylase (HDAC) inhibitor that increases histone acetylation, leading to a more open chromatin structure and enhanced gene expression.
VPA	Valproic acid—a short-chain fatty acid and HDAC inhibitor that alters chromatin structure and gene expression, widely used in neurological disorders and studied for epigenetic modulation.
**Cell lines**	
IMR-32	A human neuroblastoma cell line characterised by MYCN amplification and frequently used in cancer and neurodevelopmental research.
LAN-1	A human neuroblastoma cell line with MYCN amplification, often used to study tumour aggressiveness and therapeutic responses.
Neuro-2a (N2a)	A mouse neuroblastoma cell line widely used as a model for neuronal differentiation, signalling, and cytotoxicity studies.
NGP	A human neuroblastoma cell line with MYCN amplification, commonly used in studies of oncogene function and targeted therapies.
SH-SY5Y	A human neuroblastoma cell line (subclone of SK-N-SH) frequently used as a model for neuronal function, differentiation, and neurodegenerative diseases.
SK-N-BE	A human neuroblastoma cell line (including subtypes like SK-N-BE(2)) known for MYCN amplification and use in high-risk neuroblastoma research.
BE(2)-M17	A subclone of the SK-N-BE lineage derived from human neuroblastoma, commonly used in studies of neuronal differentiation and neurobiology.
SK-N-SH	A human neuroblastoma cell line that gives rise to subclones such as SH-SY5Y, used in neurobiology and cancer studies.
SMS-KCNR	A human neuroblastoma cell line with MYCN amplification, often used in studies of tumour biology and drug response.
TET-21/N	A human neuroblastoma cell line engineered with tetracycline-regulated MYCN expression, allowing controlled studies of MYCN function.
